# Fabrication of Graphite Flake/Al Composites via the Hybrid Powder-Melt Process: Synergistic Enhancement of Strength and Conductivity Through Low Content Addition

**DOI:** 10.3390/ma18204683

**Published:** 2025-10-13

**Authors:** Jiapeng Luo, Chunyang Lu, Feihua Liu, Xinwei Yang, Ziren Wang, Qian Qian, Ming Yan, Haihui Lin

**Affiliations:** 1 Key Laboratory of Energy Conversion Optoelectronic Functional Materials of Jiangxi Education Institutes, School of Mathematics and Physics, Jinggangshan University, Ji’an 343009, China; 2 Shinemax Advanced Materials Co., Ltd., Shenzhen 518000, China; 3 Department of Materials Science and Engineering, Southern University of Science and Technology, Shenzhen 518055, Chinayanm@sustech.edu.cn (M.Y.); 4 School of Integrated Circuits, Harbin Institute of Technology, Shenzhen 518055, China; liufeihua@hit.edu.cn; 5 State Key Laboratory of Chemistry and Utilization of Carbon Based Energy Resources, College of Chemistry, Xinjiang University, Xinjiang 830017, China; 6 National Key Laboratory of Aerospace Flight Technology, Beijing 100074, China; 7 Jiaxing Research Institute, Southern University of Science and Technology, Jiaxing 314031, China

**Keywords:** graphite flake/Al composite, electrical conductivity, tensile strength, microstructure, secondary remelting

## Abstract

This study addresses the challenge of simultaneously improving the electrical conductivity and strength of aluminum alloys. We innovatively combine powder metallurgy with melt stirring casting to fabricate graphite flake-added aluminum matrix composites through secondary remelting, electromagnetic stirring, and extruding. The influence of graphite flake content gradient (0–3.0 wt.%) on the mechanical properties and electrical conductivity was systematically investigated. Our results demonstrate that the composite with 0.2 wt.% graphite flakes (sample GM02) exhibits optimal comprehensive performance: tensile strength reaches 100.9 MPa (a 124% increase over pure Al), and electrical conductivity reaches 67.1% IACS (a 9.6% increase). Microstructural analysis reveals that low-content graphite flakes effectively suppressed electron scattering by forming semi-coherent interfaces. However, when graphite flake content exceeds 0.5 wt.%, a significant decrease in conductivity and plasticity (elongation below 10%) occurs due to increased Al_4_C_3_ phase formation, enhanced grain boundary scattering caused by grain refinement, and porosity defects induced by graphite flake agglomeration. This study provides a novel approach for the industrial production of high-performance, lightweight conductive components.

## 1. Introduction

Pure aluminum (Al) is widely used in power transmission and distribution systems (e.g., wires, cables, and busbars) due to its excellent electrical conductivity [1]. Although its conductivity is only ~60% of that of copper, it offers advantages over many common metals. Additionally, pure Al possesses low density and cost, enabling lightweight and cost-effective power systems. Aluminum alloys also generally exhibit superior corrosion resistance, maintaining good performance in harsh environments. However, their tensile strength (~40–85 MPa) is significantly lower than that of copper conductors.

With the development of new energy vehicles, replacing part of the copper wiring with aluminum alloys has become an important trend. To enhance the mechanical properties of aluminum alloys, strategies such as Severe Plastic Deformation (SPD) [2,3,4], alloying (the samples involved did not undergo additional furnace heat treatment after forming) [5,6], and heat treatment [7,8] have been employed. SPD refines grains, forming ultrafine-grained structures that significantly improve mechanical properties. For instance, spherical cavity equal channel angular expansion extrusion can produce a relatively uniform submicron structure in pure Al, increasing hardness by 91.75% compared to the as-cast state [2]. Adding 7.3 wt.% Y increased the tensile strength of pure Al by 250% [5]. By adding small amounts of Fe and Cu, an aluminum alloy conductor achieved a tensile strength of 135 MPa while retaining high conductivity (60.6% IACS) [6]. The Al-Mg-Si system is a typical age-hardenable alloy, where appropriate heat treatment increases tensile strength with minimal loss of electrical conductivity [7].

However, these strengthening strategies share a common limitation: electrical conductivity typically decreases significantly with increasing strength, making it difficult to achieve high strength while maintaining or enhancing conductivity. Like other metals, electrical conduction in Al relies on the movement of free electrons. After SPD, high-density dislocations and fine grains impede the directional movement of electrons, reducing the mean free path and mobility, thus lowering conductivity [3,9]. Solid solution heat treatment accelerates the diffusion of alloying elements into the matrix, increasing lattice distortion and electron scattering, leading to higher resistivity [10,11].

To balance conductivity and strength in aluminum alloys, graphene/Al and graphite flake/Al composites (Grf/Al composites) have been developed. Graphene, an exceptional material, is often used as an additive to enhance electrical and thermal conductivity. Graphene boasts an electrical conductivity of up to 2272% IACS, thermal conductivity exceeding 5300 W/(m·K), and outstanding mechanical properties (Young’s modulus ~1 TPa, fracture strength ~130 GPa) [12]. In parallel to its application in metals, graphene and its derivatives like graphite nanoplatelets (GNPs) have also demonstrated significant potential in polymer matrix composites. For instance, Liu et al. [13] and Mingione et al. [14] reported that the incorporation of GNPs improved the heat resistance, electrical conductivity, and tribological properties of polymers, respectively. Regarding metal matrix composites, there have also been numerous reports on incorporating graphene. For example, Chyada et al. [15] fabricated graphene/Al composites via melt casting, achieving a conductivity of 36.8 MS/m (63.4% IACS) and a tensile strength of 180 MPa after 90% cold rolling and aging at 473 K for 1 h. The aging process formed second phases and dislocation cells, reducing lattice distortion and thereby increasing conductivity. Zhou et al. [16] produced graphene/Al composites via spark plasma sintering (SPS) with 60% IACS conductivity. They observed the formation of single-crystal Al_4_C_3_ nanorods at a sintering temperature of 883 K, leading to strong interfacial bonding without significant interfacial sliding, thus enhancing composite strength. The unique interfacial reaction between graphene and the Al matrix enables effective load transfer while maintaining conductivity close to the matrix. Current primary methods for preparing highly conductive graphene/Al composites include powder metallurgy (PM) and melt stirring casting. PM involves ball milling, mixing, compaction, and sintering to uniformly disperse graphene in the Al matrix, achieving high conductivity. Aditya Nittala et al. [17] used a modified melt stirring casting method, adding 0.25% graphene modifier to increase composite conductivity from 60.3% IACS to 61.96% IACS while improving interfacial bonding strength. Despite significant progress in graphene/Al composites [15,16,17,18,19,20,21,22,23], the mechanism underlying conductivity enhancement, particularly at low graphene contents, requires further elucidation.

This study proposes a hybrid process combining PM and melt stirring casting to fabricate graphite flake/Al composites (Grf/Al composites) with conductivity reaching 67.1% IACS. We investigate the variation of mechanical properties and electrical conductivity with graphite flake content, analyze the relationship between microstructure and properties, and elucidate the structure–property correlation induced by adding graphite flake to pure Al.

## 2. Materials and Experiments

### 2.1. Raw Materials and Fabrication Process

Commercial pure aluminum powder (CP-Al, >99.8%, D_50_ = 5 μm, supplied by Henan Yuanyang Powder Technology Co., Ltd., Changyuan, China) and high-purity commercial multi-layer graphite flake (Grf) were used as primary raw materials. The composition of the Al powder is listed in Table 1. Polyamide (PA) powder (D_50_ = 15 μm) served as a binder, and anhydrous ethanol as the solvent for mixing Al powder and Grf. The Grf had a purity of 99%, oxygen content below 500 ppm, moisture content below 800 ppm, flake thickness approximately 50 nm, and flake diameter D_50_ = 800 nm (supplied by Donghua Advanced Materials (Anhui) Co., Ltd, Hefei, China). Anhydrous ethanol (1.2 kg) and PA powder (0.01 kg) were sequentially added to a stirred drying kettle and stirred for 120 min for dissolution. The Grf (0.3 kg) was then added, and the mixture was stirred for another 120 min to ensure uniform dispersion. Subsequently, Al powder (5.7 kg) was added, and the mixture was stirred at 45 rpm for 120 min under vacuum. The mixed slurry was dried at 393 K under low vacuum (absolute pressure < 20 Pa) for 12 h. The dried powder blend was packed into a polyurethane can, vibrated on an electromagnetic vibrating table for compaction, and vacuum-sealed. Cold isostatic pressing (CIP) was performed at 150 MPa for 4 min to form the initial Grf/Al master alloy billet. The green compact was sintered in a vacuum furnace at 853 K for 160 min, followed by furnace cooling. The sintered billet was extruded into a rod-shaped master alloy with a diameter of 9.5 mm, yielding a Grf/Al master alloy with 5 wt.% nominal carbon content.

**Figure 1:**
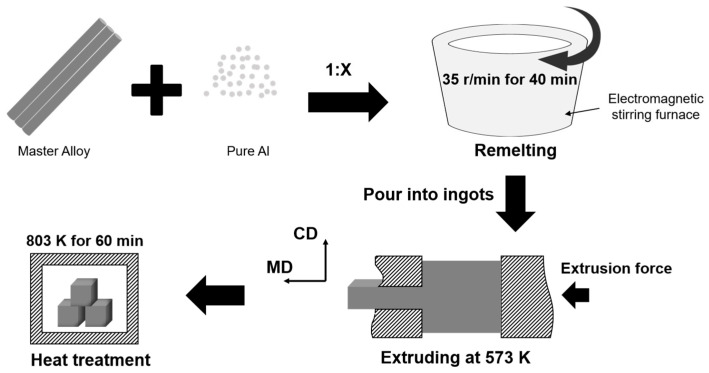
Schematic diagram of the hybrid powder-melt process for fabricating graphite flake/Al composites.

The master alloy underwent secondary remelting. The process flow is shown in Figure 1. A rod-shaped master alloy was added to molten pure Al in ratios of 1:X (specific ratios in Table 2) and electromagnetically stirred at 35 rpm for 4 min. After slag removal and degassing at 1003 K, the melt was cast. The Grf/Al rods were induction-heated to 573 K and extruded into rods with a 10 mm × 10 mm cross-section. All rods were solution-treated at 803 K for 60 min, rapidly cooled to room temperature. In addition, pure Al (GM00), fabricated identically to composites (CP-Al powder without Grf addition), underwent identical processing: powder mixing (without Grf), CIP, sintering, extrusion, remelting, and heat treatment.

### 2.2. Performance Testing

Tensile specimens were cut from composites with different Grf contents. Quasi-static uniaxial tensile tests were performed at room temperature on an INSTRON 68TM-30 universal testing machine (Instron, Electromechanical & Industrial Products Group, Norwood, MA, USA) with a gauge length of 25 mm and a strain rate of 3 × 10^−4^ s^−1^. To ensure reproducibility, a minimum of two valid tests were conducted for each composite material. Vickers hardness (HV) was measured using a fully automatic hardness tester (Emcotest/DuraScan70G5, Swiss Dantsin Technology Co., Ltd., Lausanne, Switzerland) with a load of 49.03 N (5 kgf) and a dwell time of 15 s. Ten measurements were taken per sample, and the average value was used. Electrical conductivity was measured at 293 K using a Xiamen Tianyan Smart conductivity tester (Xiamen Tianyan Instrument Co., Ltd, Xiamen, China). The instrument was calibrated before each measurement, with a precision of ±0.3%. Conductivity is reported as a percentage of the International Annealed Copper Standard (% IACS = σ_Al_/σ_Cu_ × 100%), where σ_Al_ is the conductivity of the Al alloy in MS/m and σ_Cu_ is the conductivity of copper at 20 °C (58.0 MS/m).

### 2.3. Microstructural Characterization

Typical samples (focusing on those with excellent or anomalous performance) were selected for microstructural characterization using X-ray diffraction (XRD), scanning electron microscopy (SEM) equipped with energy dispersive spectroscopy (EDS) and electron backscattered diffraction (EBSD), and transmission electron microscopy (TEM). Phase analysis was performed using a Rigaku Smartlab X-ray diffractometer (Rigaku Corporation, Tokyo, Japan) with Cu Kα radiation (λ = 1.54059 Å), operating at 40 kV and 35 mA. Scans ranged from 10° to 90° at a rate of 5°/min with a step size of 0.02°.

Samples for SEM (secondary electron imaging, backscattered electron imaging, EDS) and EBSD were ground and polished to 3000 grit, followed by vibratory polishing with 0.05 μm SiO_2_ suspension for 4 h. SEM observation and EDS elemental mapping were conducted using a ZEISS Merlin field-emission scanning electron microscope equipped with an EDAX spectrometer and EBSD detector (Merlin, ZEISS, Baden-Wurttemberg, Germany). The EBSD scan step size was 0.08 μm. To observe the Grf/Al interface structure and flake orientation in detail, TEM analysis was performed using a TECNAI G2F30 microscope (FEI Company, Hillsboro, OR, USA). TEM samples were mechanically thinned to 80 μm and then finally thinned using a Gatan PIPS II 695 precision ion polishing system (Gatan, Inc., Pleasanton, CA, USA).

## 3. Results and Discussion

### 3.1. Properties of Graphite Flakes/Al Composites

**Figure 2:**
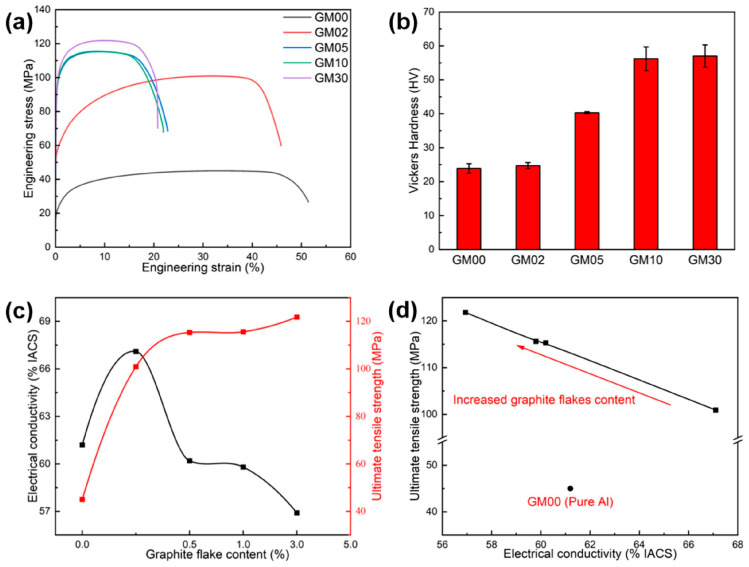
(**a**) Engineering tensile stress–strain curves of pure Al (GM00) and graphite flake/Al composites; (**b**) Vickers hardness of the composites as a function of graphite flakes content; (**c**) Variations of electrical conductivity and tensile strength with graphite flakes content, the black line represents the relationship between graphite flake content and electrical conductivity, while the red line represents the relationship between graphite flake content and tensile strength; (**d**) Relationship between electrical conductivity and tensile strength, the red arrow indicates the trend of increasing graphite flake content.

The mechanical properties and electrical conductivity of composites (representative samples) with different Grf contents are summarized in Figure 2. Table 3 shows the test averages and standard deviations. Compared to pure Al (GM00), adding Grf significantly increased tensile strength and hardness but reduced ductility. Typical engineering stress–strain curves are shown in Figure 2a. This trend aligns with the general rule of reinforcement addition in metal matrices and existing literature [16,17,19]. With increasing Grf content (0–3.0 wt.%), tensile strength and Vickers hardness increased monotonically (Figure 2b), while uniform elongation continuously decreased (Figure 2a). Notably, when Grf content exceeded 0.5 wt.%, the strength increase slowed significantly (only 5.6% increase from GM05 to GM30), while elongation remained low (<10%). Crucially, the GM02 sample with 0.2 wt.% Grf exhibited exceptional comprehensive properties: tensile strength (100.9 MPa) increased by 124% compared to pure Al (GM00, 45.0 MPa), while uniform elongation only decreased slightly to 32.9% (approximately 5.7% decrease). In contrast, while GM05 (0.5 wt.%) showed a further 14.3% increase in tensile strength (115.3 MPa) over GM02, its uniform elongation plummeted to 9.5% (a 70.8% decrease). The trend in Vickers hardness (Figure 2b) mirrored tensile strength, increasing with Grf content, contrasting sharply with the decreasing elongation. This trend is also consistent with the series of experiments conducted by Pérez-Bustamante et al. [24]. Thus, a slight addition of Grf (0.2 wt.%) significantly strengthens pure Al while maintaining good plasticity.

Electrical conductivity exhibited a non-monotonic trend with Grf content (Figure 2c,d). GM02 achieved the maximum conductivity of 67.1% IACS, a significant 9.6% increase over pure Al (GM00, 61.2% IACS). However, conductivity dropped sharply when Grf content exceeded 0.5 wt.% (GM05: 60.2% IACS) and decreased further with higher content (GM30: 56.9% IACS). This deterioration trend aligned with the decrease in elongation. As shown in Figure 2d, conductivity and tensile strength were negatively correlated across the samples: higher conductivity corresponded to lower strength. Remarkably, only GM02 (0.2 wt.%) achieved both high strength (a 124% increase) and higher conductivity than the pure Al matrix (a 9.6% increase). While previous work by Atanacio-Sánchez et al. [7] demonstrated strength improvement without compromising conductivity, the present study successfully achieved a synergistic enhancement of both properties. In summary, the GM02 sample, fabricated via the hybrid powder-melt process, successfully achieved a synergistic enhancement of strength and conductivity (Figure 2c), demonstrating great potential as a high-performance lightweight conductive material (e.g., for wires).

A comparative analysis of the mechanical and electrical properties of various Al-based composites from this work and the literature is summarized in Table 4. It is evident that the GM02 composite (0.2 wt.% Grf/Al) developed in this work exhibits a superior combination of tensile strength (100.4 MPa) and electrical conductivity (67.1% IACS). While some powder metallurgy routes employing ball milling can achieve exceptionally high strength [18], this often comes at a significant cost to conductivity (e.g., 51% IACS) due to severe lattice defects introduced during intensive mechanical mixing. Conversely, processes such as continuous casting [19] and semi-solid extrusion [25] achieve appreciable gains in strength with only a minimal compromise to electrical conductivity. Notably, the significant conductivity deterioration observed in Al2219-graphene composites [26] at higher filler content underscores the challenge of dispersion and interface control. In contrast, our hybrid powder-melt process successfully fabricates a Grf/Al composite that not only significantly strengthens pure Al but also uniquely enhances its conductivity, demonstrating a remarkable synergy that is rarely achieved in metal matrix composites.

### 3.2. Distribution of the Graphite Flake

**Figure 3:**
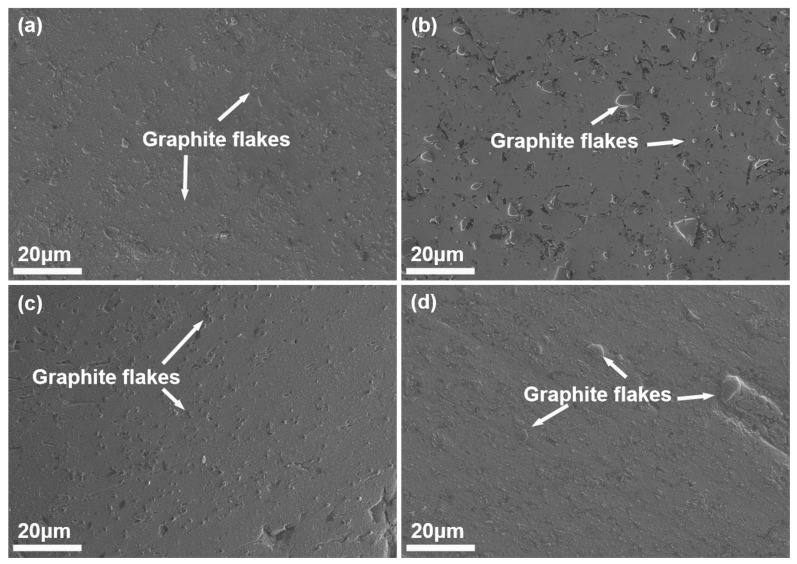
SEM micrographs (machine direction cross-section) showing the distribution of graphite flakes in composites with different graphite flakes contents: (**a**) GM02: 0.2 wt.%; (**b**) GM05: 0.5 wt.%; (**c**) GM10: 1.0 wt.%; (**d**) GM30: 3.0 wt.%. Dark regions: Graphite flakes. Note: Al_4_C_3_ phases (<100 nm) are not resolved at this magnification.

**Figure 4:**
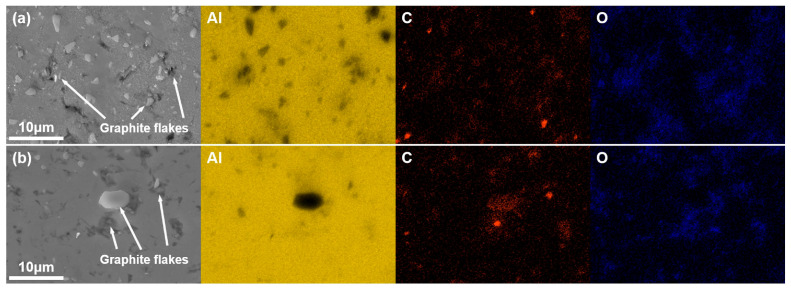
EDS elemental mapping (machine direction cross-section) of (**a**) GM02 and (**b**) GM05 samples: showing the distribution of Al, C, and O elements.

**Figure 5:**
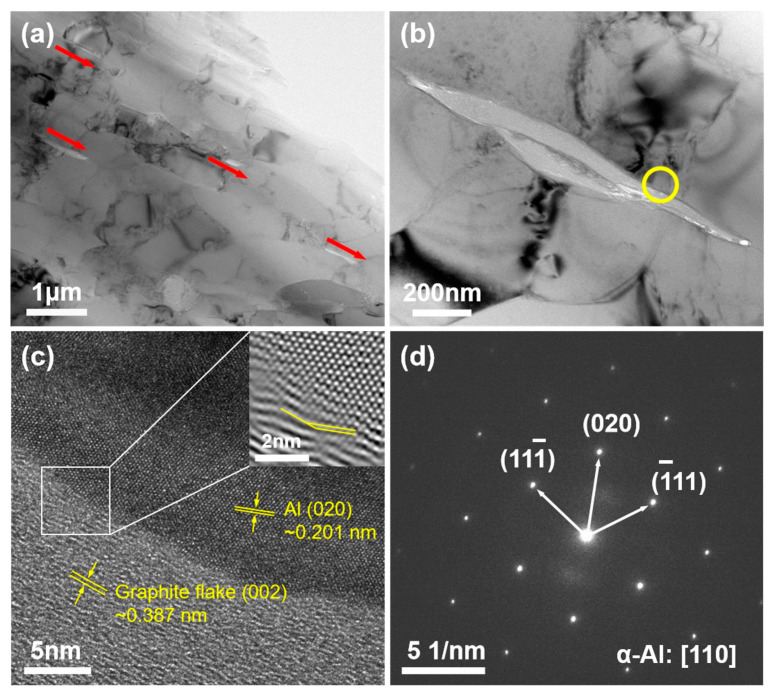
TEM characterization of the GM02 sample: (**a**) Low-magnification TEM image showing the orientation of Graphite flakes sheets parallel to the machine direction (ED); (**b**) TEM image showing the Graphite flakes/Al interface; (**c**) HRTEM image of the interface region marked by the yellow circle in (**b**) (inset: FFT pattern from the white square region); (**d**) SAED pattern taken from the yellow circled area in (**b**).

SEM micrographs (Figure 3) and EDS elemental maps (Figure 4) reveal the distribution of Grf within the Al matrix. EDS elemental maps (Figure 4) quantitatively confirm the homogeneous C distribution in GM02 (0.2 wt.%), whereas localized C-rich agglomerates (>5 μm) dominate in GM05 (0.5 wt.%). Figure 4 shows that the matrix is primarily Al. The Grfs (corresponding to C-rich regions) are distributed within the matrix. Notably, significant oxygen (O) enrichment was observed in Grf agglomeration areas (Figure 4b), suggesting that oxides (e.g., Al_2_O_3_) tend to form at or near the Grf/Al interface. The observed oxygen primarily originates from the sample preparation process, where porous regions at Grf agglomerates and Grf/Al interfaces likely trapped air oxygen, leading to accelerated surface oxidation of graphite flakes and consequent oxygen enrichment. However, the amount of enriched oxygen is extremely small compared to the added Grf, and its contribution to the mechanical properties is considered negligible. Therefore, the subsequent discussion on the enhancement of mechanical properties will primarily focus on the role of the Grf addition itself. Figure 3a shows that in the low-content sample GM02 (0.2 wt.%), Grf (dark strips/flakes) are relatively uniformly distributed with no obvious agglomeration, largely retaining their initial size (600–1000 nm). Simultaneously, some Grfs fractured during secondary remelting and subsequent processing, forming smaller fragments (less than 500 nm) embedded in the matrix. The Al_4_C_3_ phases formed by interfacial reactions are generally sub-100 nm in size [23,27] and thus not visible at this scale. With increasing Grf content (Figure 3b–d), the dispersion efficiency of electromagnetic stirring decreased, leading to significant Grf agglomeration and stacking. Large Grf aggregates up to 10 μm were clearly observed in higher-content samples (GM05, GM10, and GM30). TEM observation (Figure 5a) further confirmed that in the GM02 sample, Grfs tended to align parallel to the machine direction (MD).

**Figure 6:**
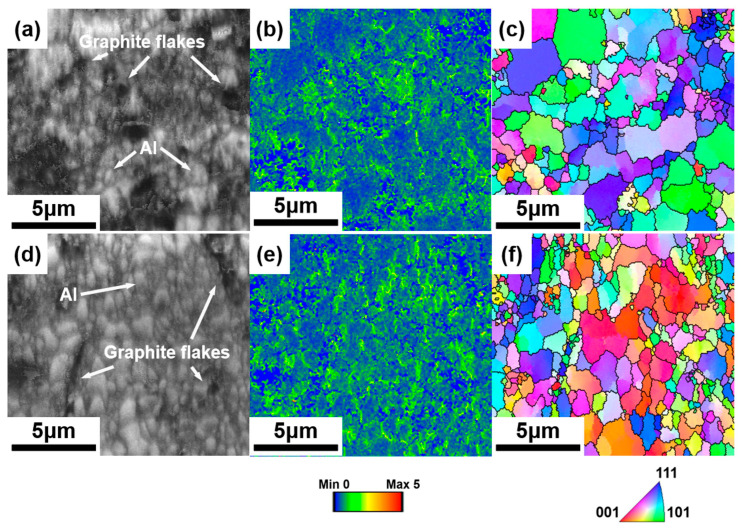
EBSD analysis of GM02 and GM05 samples: (**a**,**d**) Image quality (IQ) maps; (**b**,**e**) kernel average misorientation (KAM) maps; (**c**,**f**) inverse pole figure (IPF) maps along the machine direction (MD).

To further observe microstructural details, Figure 6 presents Electron Backscattered Diffraction (EBSD) images and results for samples GM02 and GM05, exhibiting the largest performance gap. Figure 6a,d show Image Quality (IQ) maps; the Grfs appear black as they are non-crystalline. Combining Figure 6b,e (Kernel Average Misorientation-KAM maps) reveals that deformation occurred in the Al matrix surrounding Grf, increasing local dislocation density. Larger Grfs, primarily located at grain boundaries, induced larger strain, while smaller Grfs within grains caused much less strain, as indicated by KAM. Figure 6c,f (Inverse Pole Figure-IPF maps) show that grain sizes near large Grfs are much smaller than elsewhere, confirming that Grf addition significantly refined the grains in the composites.

### 3.3. Effect of the Graphite Flakes on Microstructure

**Figure 7:**
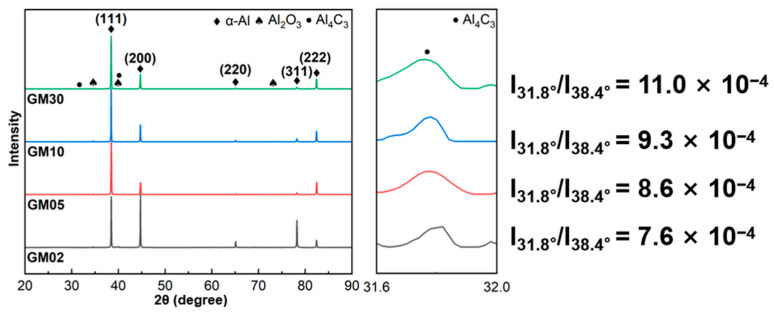
XRD patterns of graphite flakes/Al composites with different graphite flakes contents (machine direction cross-section). The inset shows the relative intensity of the Al_4_C_3_ peak.

Figure 7 shows XRD patterns of the composites’ cross-sections along the machine direction for different Grf contents. The relative intensity of the Al (220) diffraction peak in GM02 is significantly higher than in other samples, while its Al (111) peak intensity is similar to high-content samples. This indicates a stronger {111}<110> texture in GM02. For face-centered cubic (FCC) Al, {111}<110> is the primary slip system; a stronger texture of this orientation generally enhances plastic deformation capability. This may be a factor contributing to GM02’s superior ductility. Furthermore, XRD detected the presence of the Al_4_C_3_ phase in all Grf-containing samples (Figure 7), a product of the interfacial reaction between Grf and the Al matrix. As a brittle phase, Al_4_C_3_ pins dislocation motion, which is one of the important factors leading to the decrease in the plasticity of composite materials. Calculating the relative intensity of the characteristic Al_4_C_3_ diffraction peak (Figure 7 inset) shows it increases monotonically with Grf content, indicating more Al_4_C_3_ phase formation. This correlates with the decreasing trend in plasticity (elongation). Additionally, Al_4_C_3_ primarily precipitates at grain boundaries [16,27]; its increased content indirectly enlarges the total grain boundary area and dislocation density (as analyzed below), which scatters electron transport, leading to decreased conductivity.

**Figure 8:**
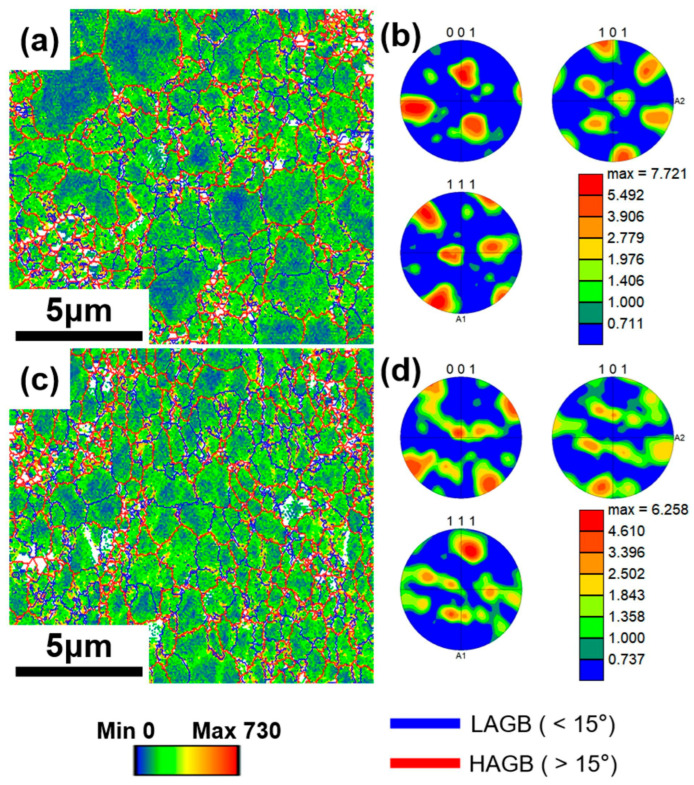
(**a**,**c**) Geometrically necessary dislocation (GND) density maps overlaid on orientation maps for GM02 and GM05, respectively; (**b**,**d**) pole figures (PF) for GM02 and GM05, respectively.

**Figure 9:**
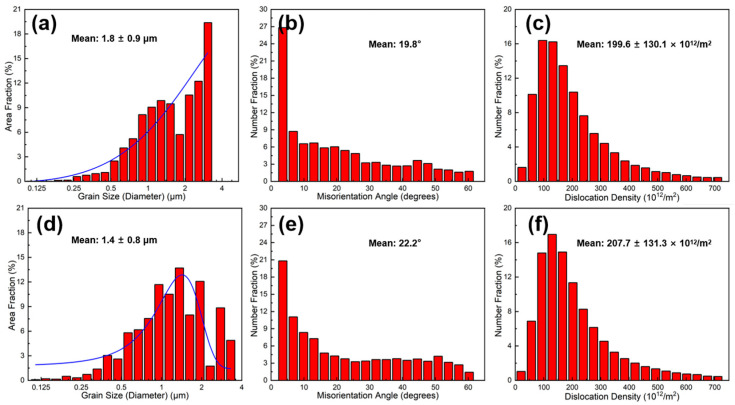
Statistical analysis of EBSD data for GM02 (**a**–**c**) and GM05 (**d**–**f**): (**a**,**d**) Grain size distribution; (**b**,**e**) misorientation angle distribution; (**c**,**f**) GND density distribution.

Based on KAM maps obtained from EBSD (Figure 6b,e), Geometrically Necessary Dislocation (GND) density maps (Figure 8a,c) and their statistical distributions (Figure 9c,f) were calculated for GM02 and GM05. Figure 8a,c clearly show significant dislocation enrichment zones around Grf nanoplates. Statistical analysis (Figure 9c,f) indicates that the average GND density of GM05 (0.5 wt.%, 207.7 × 10^12^ m^−2^) is slightly higher than that of GM02 (0.2 wt.%, 199.6 × 10^12^ m^−2^). The introduction of Grf not only increased dislocation density but also increased misorientation angles between grains. Grain boundary misorientation distribution statistics (Figure 9b,e) show that the proportions of low-angle grain boundaries (LAGB, misorientation < 15°) in GM02 and GM05 are 49.4% and 48.2%, respectively, while the proportions of high-angle grain boundaries (HAGB, misorientation ≥ 15°) are 50.6% and 51.8%, respectively. Grain size distribution statistics (Figure 9a,d) confirm the significant grain refinement effect of Grf. The average grain size of GM05 (0.5 wt.%, 1.4 μm) is notably smaller than that of GM02 (0.2 wt.%). Non-normal distribution observed in GM02 (Figure 9a) likely results from localized severe grain refinement induced by fragmented Grf flakes in specific zones, concurrent with insufficient refinement in adjacent regions where Grf density was low. This distribution deviates from a normal distribution due to localized variations in Grf density. Regions with high Grf concentration (e.g., near fragmented flakes) experience severe grain refinement, while adjacent areas with low Grf density retain larger grains. Extruded pure Al typically forms strong textures. However, Grf addition significantly inhibited texture formation and development (Figure 8b,d). Grf particles (especially agglomerates), their interfaces (e.g., Grf/Grf and Grf/Al), and potential pores hindered grain rotation and preferential orientation growth during extruding. Consequently, with increasing Grf content, dispersion became more difficult, agglomeration worsened, leading to significantly weaker texture intensity in composites like GM05 compared to low-content GM02 (compare Figure 8b,d). Figure 8b,d show Pole Figure (PF) maps for GM02 and GM05. At 0.2 wt.% Grf, a {110}<100> texture is still present. When Grf content increased to 0.5 wt.%, no distinct texture was observed, indicating reduced orientation preference.

### 3.4. Effect of the Graphite Flakes on Properties

The strengthening mechanisms in Grf/Al composites typically include synergistic effects of Orowan bypassing, grain boundary strengthening (grain refinement), and second-phase strengthening [28,29,30]. Orowan strengthening originates from nano-scale hard particles (e.g., fragmented Grf, Al_4_C_3_) obstructing dislocation motion, forcing dislocations to bow out and bypass the particles, requiring higher applied stress. In this study, some Grf fractured into nanoflakes (<100 nm) during secondary remelting and processing. Even at low volume fractions (<1%), the Orowan mechanism contributed significantly to strength enhancement [31]. Additionally, the nano-scale Al_4_C_3_ phase formed by the interfacial reaction acts as second-phase strengthening points, hindering dislocation motion. Concurrently, as previously described (Figure 9a,d), Grf significantly refined grains, contributing to strength via the Hall–Petch relationship. And the Grf aligned parallel to the tensile direction (i.e., machine direction) may partially bear tensile stresses (as shown in Figure 5a), contributing to load transfer strengthening. Therefore, the monotonic increase in tensile strength with Grf content (up to GM05) resulted from the combined increase in nano-Grf flake density, Al_4_C_3_ phase content, and continuous grain refinement. However, severe agglomeration induced by excessive Grf (>0.5 wt.%) caused porosity defects (Figure 3c,d), which not only drastically deteriorated plasticity but also acted as stress concentration sites, limiting further strength gains (GM05–GM30 strength plateau). Strengthening is partly attributed to work hardening from geometrically necessary dislocations (GNDs) pinned by Grf (Figure 8a,c). Furthermore, Orowan strengthening and grain refinement effects approach their maximum efficiency at 0.5 wt.% Grf. As evidenced by stabilized dislocation density (Figure 9f) and grain size (Figure 9d) in higher-content samples, further Grf addition cannot significantly enhance these strengthening mechanisms, resulting in the observed strength plateau. This indicates an optimal/critical Grf content (0.2–0.5 wt.%) exists for maximum strengthening.

Composite conductivity is influenced by multiple factors, including interface density (grain boundaries and phase boundaries), interface properties (orientation, coherency, and impurities), dislocation density, and defects like porosity. As mentioned, grain refinement (Figure 9a,d) inevitably increases the total grain boundary length while enhancing strength. Grain boundaries act as electron scattering centers; increased density significantly reduces conductivity, especially when grain sizes approach the nanometer scale [32]. Furthermore, boundary type (misorientation) significantly affects resistivity: Low-Angle Grain Boundaries (LAGB) typically scatter electrons less than random High-Angle Grain Boundaries (HAGB). Specifically, coherent twin boundaries exhibit much lower resistivity than general HAGB. Therefore, introducing a high proportion of twin boundaries can strengthen the material while maintaining high conductivity [33]. In this study, with increasing Grf content: (1) Grain size decreased (more scattering centers); (2) texture weakened (Figure 8b,d), leading to more random grain orientations; and (3) the HAGB proportion slightly increased (Figure 9b,e: GM02: 50.6% vs. GM05: 51.8%). These three factors collectively caused stronger electron scattering at grain boundaries, primarily responsible for the significant conductivity drop when Grf content exceeded 0.5 wt.%. Additionally, the disordered Grf/Al interfaces, Grf/Grf interfaces, and porosity defects (Figure 3c,d) introduced by high-content Grf agglomeration further intensified electron scattering, severely impairing conductivity. Crucially, only the low Grf content (0.2 wt.%) GM02 sample exhibited conductivity (67.1% IACS) surpassing the pure Al matrix (61.2% IACS). TEM analysis (Figure 5b) revealed that at this content, Grf formed clean, well-bonded interfaces with the Al matrix. High-resolution TEM (HRTEM) images (Figure 5c) and Selected Area Electron Diffraction (SAED) patterns (Figure 5d) provided atomic-scale details of the interface structure. The measured interlayer spacing of adjacent Grf flakes was 0.387 nm, close to the (002) plane spacing of graphite [34]. The inset FFT pattern and HRTEM in Figure 5c show direct contact between the Grf (002) plane and the adjacent Al matrix (020) plane. The measured misorientation angle between them was 19.9°, close to the average grain boundary misorientation in that region (19.8°). More importantly, no amorphous layer or significant impurities were observed at the interface, and the lattice exhibited a degree of continuity across the interface (Figure 5c), indicating the formation of a low-defect-density semi-coherent interface structure. The interfacial mismatch was calculated as δ = 0.038 using the formula δ = (2(d_β_ − d_α_))/(d_β_ + d_α_), where d_α_ = 0.387 nm is the interlayer spacing of graphite flakes and d_β_ = 0.402 nm is the d-spacing of Al(010) derived from Al(020). This value confirms a semi-coherent interface (δ < 0.25). These clean, low-resistance semi-coherent interfaces, combined with the extremely high intrinsic conductivity of Grf, provided efficient, low-scattering electron transport pathways. This is identified as the key microstructural mechanism enabling GM02’s conductivity to exceed that of pure Al.

To briefly conclude, this study elucidates the micro-mechanism of synergistic enhancement of strength and conductivity in Al matrix composites by slight Graphite flakes, particularly highlighting the critical role of semi-coherent interfaces. Furthermore, the developed hybrid powder-melt process (HP-MP) holds significant potential for industrial application, providing a novel technical pathway for manufacturing high-strength, high-conductivity, lightweight conductive components (e.g., wires and busbars) urgently needed in fields such as new energy vehicles and low-altitude economies.

## 4. Conclusions

This study successfully developed a hybrid process combining powder metallurgy with secondary remelting and electromagnetic stirring (HP-MP), enabling the industrially feasible fabrication of high-performance Grf/Al composites. At a Grf addition level of 0.2 wt.%, the composite (GM02) exhibited outstanding comprehensive properties: tensile strength reached 100.9 MPa, a remarkable 124% increase over the pure Al matrix; simultaneously, electrical conductivity reached 67.1% IACS, a 9.6% increase over the matrix. Microstructural characterization and mechanistic analysis revealed the following:

1. Slight Grf (0.2 wt.%) can be uniformly dispersed via the HP-MP, forming clean, low-resistance semi-coherent interfaces with the Al matrix, which is key to conductivity enhancement.
2. Grf synergistically enhances strength through Orowan strengthening, grain refinement strengthening, and Al_4_C_3_ second-phase strengthening.
3. Excessive Grf (>0.5 wt.%) causes severe agglomeration, porosity defects, increased Al_4_C_3_ phase content, excessive grain refinement, and weakened texture. These factors collectively induce strong grain boundary/interface scattering and stress concentration, leading to a drastic deterioration in conductivity and plasticity (elongation below 10%).

## Figures and Tables

**Table 1:** Chemical composition of the commercial pure aluminum powder (wt.%).

Element	Al (wt.%)	Cu (wt.%)	Fe (wt.%)	Si (wt.%)	Balance (wt.%)
Content	99.87	0.0010	0.0763	0.0456	0.0071

**Table 2:** Graphite flakes’ nominal content and master alloy dilution ratios for composite samples.

Sample ID	Master Alloy (kg)	Pure Al (kg)	Master Alloy:CP Al	Graphite Flake Content (wt.%)
GM00	0	2	-	0.0
GM02	0.25	6	1:24	0.2
GM05	0.5	4.5	1:9	0.5
GM10	1	4	1:4	1.0
GM30	2	1.333	1:0.666 (3:2)	3.0

**Table 3:** Mechanical properties and electrical conductivity of pure Al and graphite flake/Al composites.

Sample ID	Tensile Strength (MPa)	Uniform Elongation (%)	Vickers Hardness (HV)	Electrical Conductivity (% IACS)
GM00	44.6 ± 0.4	32.9 ± 2.1	23.9 ± 1.4	61.2
GM02	100.4 ± 0.5	31.6 ± 1.3	24.7 ± 0.9	67.1
GM05	115.7 ± 0.4	9.4 ± 0.2	40.3 ± 1.3	60.2
GM10	116.0 ± 0.4	8.6 ± 0.2	56.2 ± 3.5	59.8
GM30	120.9 ± 0.9	8.75 ± 0.1	57.0 ± 3.3	56.9

**Table 4:** Reported mechanical properties and electrical conductivity of graphene-reinforced Al alloy.

Composite Material	Preparation Process	Tensile Strength (MPa)	Electrical Conductivity (% IACS)	Ref.
GM00 (CP-Al)	Hybrid Powder-Melt Process	44.6	61.2	This work
GM02 (0.2 wt.% Grf/Al)		100.4	67.1	
GM05 (0.5 wt.% Grf/Al)		115.7	60.2	
Ball-milled Al	Ball milling process	(Yield strengths) 298	51	[18]
0.2 vol.% rGO/Al		(Yield strengths) 374	51	
Al	Continuous casting and rolling processes	114	62.5	[19]
Al-0.2 wt.% GNPs		156	61.8	
Al2219	Spark plasma sintering	-	50	[26]
Al2219-0.5 wt.% graphene		-	36	
Al2219-1 wt.% graphene		-	11	
Al-8030	Semisolid extrusion	130.6	62.2	[25]
0.5 wt% GNPs/Al-8030		212.7	61.9	

## Data Availability

The original contributions presented in this study are included in the article. Further inquiries can be directed to the corresponding authors.
